# The Treatment of Verrucae Pedis Using Falknor’s Needling Method: A Review of 46 Cases

**DOI:** 10.3390/jcm2020013

**Published:** 2013-04-02

**Authors:** Belinda Longhurst, Ivan Bristow

**Affiliations:** 1 Winchester Podiatry, Winchester, SO23 9QX, UK; E-Mail: belinda@winchesterpodiatry.co.uk; 2 Faculty of Health Sciences, University of Southampton, Hampshire, SO17 1BJ, UK

**Keywords:** wart, verrucae, HPV, foot, plantar, virus, infection

## Abstract

Human Papilloma Virus (HPV) related verrucae pedis persist, seemingly evading host immune surveillance, yet sometimes disappear with inflammation. The absence, or reduction, of a cellular immune response may explain why verrucae treatments are not uniformly successful and treatment can be difficult even in immune-competent individuals. Little investigation has been undertaken into the potential benefit and efficacy of needling verrucae, a treatment modality causing HPV infected keratinocyte destruction in addition to inducing an assumed enhanced immune response. A review of clinical practice is presented, reporting the treatment method and results of data collected from a retrospective review of 45 patients. Thirty-one (69%) cases demonstrated complete resolution of verrucae following needling treatment. Three patients demonstrated reduction in size and pain whilst 11 showed no improvement. No adverse events were noted. Needling may have a place in the management of verrucae pedis in an adult population but a large scale study utilising objective measures and a control intervention would provide more detailed efficacy data along with a greater understanding of the effects of this treatment on long term immunity.

## 1. Introduction

Verrucae (plantar warts) are a notorious source of frustration for both practitioners and patients alike, as no single treatment is completely effective in all patients. Despite a plethora of medical literature on this subject, high quality evidence for the efficacy of almost all treatments is non-existent [1,2]. A review by Lipke [3] also stated that although evidenced based reviews with guidelines have been published, they do not cover treatments that have yet to be subjected to blinded randomized, controlled clinical trials. Moreover, Lipke asserted that lack of robust evidence of a therapy, which has not been subjected to such rigorous scientific testing, does not mean that it is not worth knowing about nor worthy of use in practice, particularly when a specific treatment has been utilised and reported, albeit anecdotally, with a reasonably high clinical success rate. This article is a patient-centred, review of clinical practice of one such verrucae treatment, which was first described by Falknor [4] in 1969, the method of which he termed “needling”. 

Verrucae are benign tumours, caused by infection of epidermal keratinocytes by the double stranded DNA Human Papilloma Virus (HPV). There are currently more than 100 known types of HPV and these determine the anatomical distribution and morphology of the lesion [5]. The most common warts on the hands and feet are the subtypes 1, 2 and 4 [6]. 

Infection of the keratinocyte at the basal layer of the epidermis is established through abrasions of the skin surface. Here, the virus remains latent in the cell from 1 to 8 months [7]. As the epidermal cells differentiate and migrate to the surface, the virus is triggered to undergo replication and maturation until it is shed in the exfoliation of the epidermis. The process of virus replication produces proliferation of prickle cells which alters the character of the epidermis, resulting in the visible warty appearance of the verrucae.

In most viral infections, the viral proteins within a cell cause damage to the host cell and stimulate production of cytotoxic T cells, which then seek out and destroy the targeted infected cells. However, unlike many viruses, HPV prevents cell lysis as infection spreads through the shedding of infected epithelial cells from the surface of the skin. In other words, there is no (or indeed limited) release of viral proteins to the circulating dentritic cells, and therefore, no (or inadequate) antigen presentation to the immune system. Furthermore, HPV proteins also encode specific functions to inhibit immune responses by inducing specific anti-inflammatory mechanisms by activating T suppressor cells. Frazer [8] explains that “such inhibition would be expected to reduce specific antiviral defence mechanisms and also effective presentation of antigen to the host immune system”.

The absence, or reduction, of a cellular response may explain why verrucae treatments are not uniformly successful and treatment can be difficult even in immune-competent individuals. Most treatments work by destroying affected tissues, by either a cytotoxic or physically ablative mode of action. However, tissue damage alone may not be enough to produce the relevant cytokines to destroy latent virus in adjacent cells [9], thus recurrence and further treatment is often required after apparent resolution [10]. Verrucae persist, evading host immune surveillance, but sometimes disappear with inflammation [11]. Sterling *et al.* [6] stated that despite the lack of antigens, HPV does sometimes induce an immune response and spontaneous regression is often seen, although warts are less likely to resolve in adults and in immuno-suppressed patients. Therefore, research into efficacy of verrucae treatment for children must take into account the incidence of a higher rate of spontaneous regression [12]. 

Frazer’s work concluded that induction of cell-mediated immunity to early proteins of HPV may prove useful as a therapeutic approach to HPV infection [8]. This is in agreement with Tyring [13] who also stated; “The ideal way to combat HPV infection would be to improve the immune response to the virus so it is specific and directed against early viral proteins.” One way of achieving this would be by better presentation of viral antigens to the immune system. Recent research on successful treatments has been aiming toward creating an enhanced systemic immune response to eradicate the virus [7]. This enhancement is required as although HPV does induce a localised immune response, it is not effective enough to trigger a systemic response because any expression of viral proteins are limited to superficial epithelial cells, thus there is a reduced presentation of these to the immune system. 

The work of Parton and Sommerville [14] asserted that resolution of a single plantar verruca in children aged 4–14 years could be successfully achieved by lightly debriding the lesion to produce capillary bleeding and then abraded with fine glass paper. Chapman [15] expanded upon this work in 1998 and hypothesised that it should be possible to demonstrate a “whole body response” in patients with several verrucae in that the treatment of just one verruca will lead to the resolution of the untreated verrucae. Twenty-one patients participated in Chapman’s clinical trial with a wider age group range (aged 6–36 years) than that of Parton and Sommerville, who claimed an optimistic 94% of cases cleared within two weeks after one treatment. Chapman reported 43% success rate in participants aged 6–13 years. His conclusion concurred with Stirling’s observation; that treatment is more likely to be successful in patients under 14 years old. 

The ideal verrucae treatment should result in resolution of all or a great percentage of warts, be painless, need only one or a part of a lesion treated, create no scarring and offer HPV immunity for a lifetime.

Falknor [4] first explained a direct needling procedure as a form of physical trauma without the use of chemicals (notwithstanding the use of local anaesthesia). The method he outlined comprised of anaesthetising the verruca, then thrusting in a needle “in dart fashion, so as to penetrate the full depth of the verruca and exiting through the base of the capsule into the fat”. He claimed only two recurrences of 126 lesions treated with this technique. Subsequent published research using this method has been sparse. Skilton and Mehar [16] published a case series of 14 patients with painful verrucae who were treated with the needling technique. They claimed resolution in 50% (7 out of the 14) had complete resolution at 8-week review. This paper retrospectively analyses 46 cases of verruca treated using Falknor’s needling method in a single private podiatry practice. The authors present a retrospective review of 46 cases (34 female, 12 male) treated within a private practice in Hampshire, UK using Falknor’s method. 

## 2. Experimental Section

### 2.1. Methods

Ethical approval was sought through the National Research Ethics Service for this study. Each patient presented with clinically diagnosed verruca pedis. Treatment was indicated where the patient reported associated pain with the verrucae and subsequent interference with daily activities. However, a minority also requested clinical intervention as they perceived their quality of life was compromised due to cosmetic embarrassment. All treatment options, including no treatment, and the risks and benefits were explicitly explained to each patient. Those opting for needling treatment were enrolled into the study. Informed, signed consent was given by all patients prior to the needling procedure.

Data from each patient was collected at baseline (prior to the procedure), one week post-operatively and at their final review to ascertain the following:

- Previous treatment methods (at baseline only).
- Location and duration of the lesion(s) (at baseline only).
- Any discomfort throughout and at one and eight weeks post-operative (recorded as none, mild, moderate, severe).
- Any post-operative infection or scarring following the procedure (at one and eight weeks).
- Resolution or reduction in size of lesion (at eight weeks).

### 2.2. Procedure

Local anaesthetic (Mepivacaine^®^ 3%) was administered by tibial nerve block, digital block or local infiltration according to the location of the lesion chosen for needling. Once the area of skin was anaesthetised, any overlying callus was debrided. If the patient presented with mosaic or multiple plantar warts the largest and thickest lesion was selected for treatment. All treatments were undertaken by a single operator (BL).

**Figure 1:**
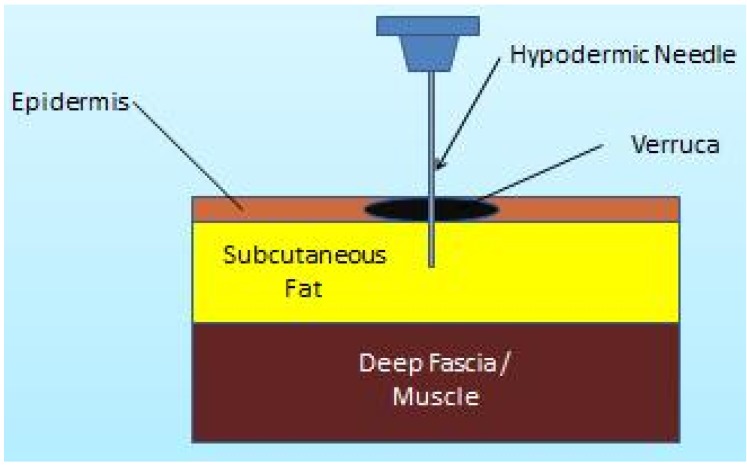
Demonstrating the needling technique.

**Figure 2:**
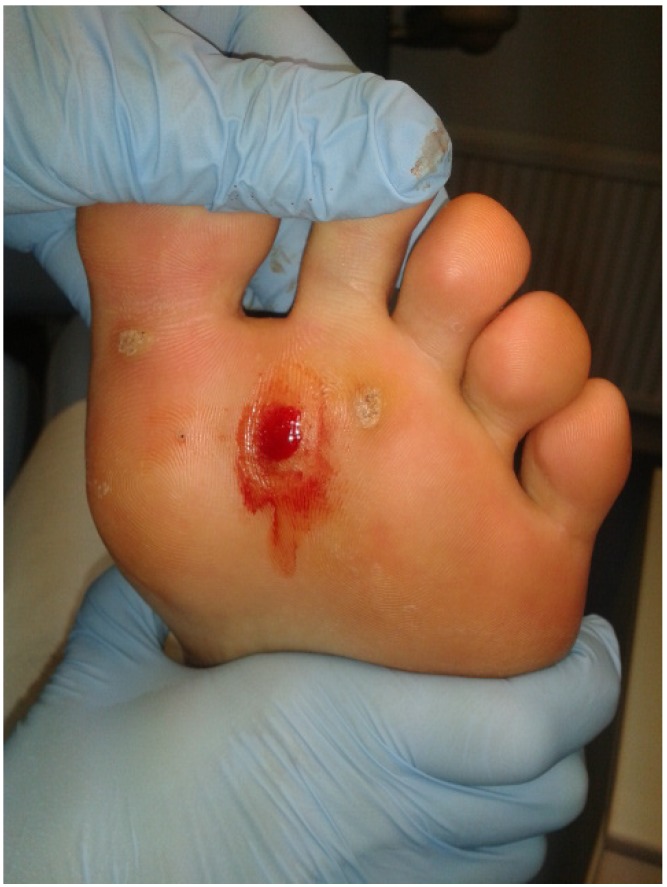
Wart. Immediately post-operatively after undergoing needling.

The area surrounding the lesion was first cleansed with povidone-iodine before an empty 27 gauge needle was utilised to puncture through the lesion to the subcutaneous tissue (Figure 1). Each puncture produced pin point bleeding and this was continued until there was no more resistance, or reactive pressure, from the epidermis and the entire lesion was perforated enough to produce a beefy red wound (Figure 2). The total number of punctures varied according to the size of the lesion. 

**Figure 3:**
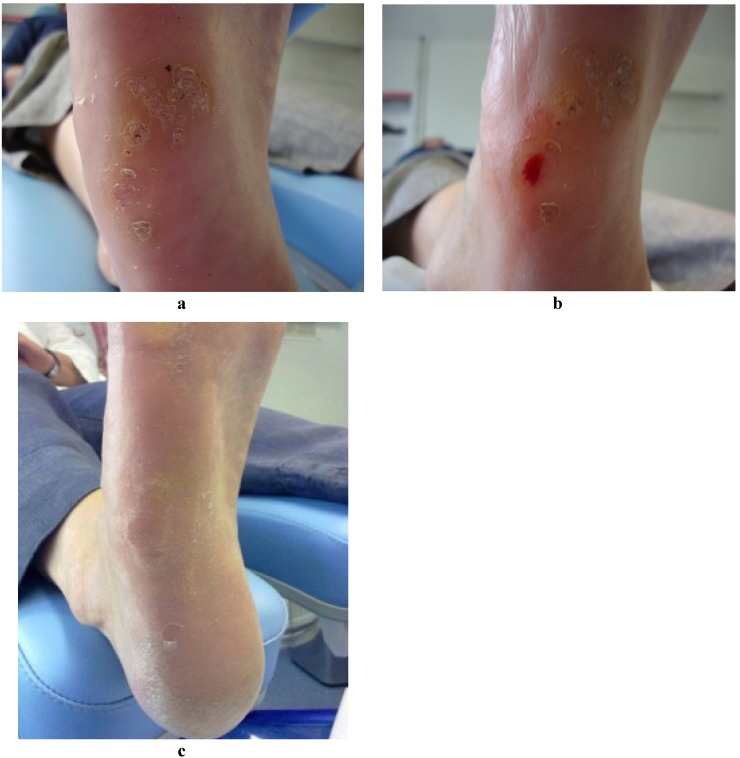
(**a**) Lateral plantar lesion before treatment; (**b**) Same lateral plantar lesion during treatment; (**c**) Same lateral plantar lesion resolved, one month after treatment.

Pressure was then applied to the wound with sterile gauze and then dressed with a non-adherent sterile dressing (Melolin^®^) and fixing tape (Mefix^®^). A semi compressed felt aperture pad was also applied on weight bearing sites to deflect pressure and reduce post-operative bruising. Each patient was issued with post-operative care sheets and advised to lightly shower and wash the area after keeping the dressing dry for 24 h. Each patient was advised to avoid taking NSAIDs or other anti-inflammatory medication for 48 h to increase the likelihood of a successful controlled inflammatory response. Wound inspection and debridement of any uncomfortable eschar was performed one week later. The final inspection for verrucae resolution was carried out 8 weeks later. Complete resolution was deemed accomplished on return of normal dermatoglyphics to the treated lesion, *i.e.*, uninterrupted skin striae and no pain on lateral compression of the area. Figure 3a–c demonstrate a lesion before, immediately after and six weeks following treatment.

## 3. Results and Discussion

A total of 46 patients (13 male, 33 female) underwent a standardised needling procedure. The mean age of the cohort was 41.8 years ± SD 12.65 (range 17–66 years). The average patient reported duration of the warts was 5.7 years ± SD 4.15 (range 1–20 years). The locations of all the treated lesions are given in Table 1. A total of 45 patients were available for review at eight weeks post-operatively with one patient lost to follow up.

**Table 1:** Location of primary (treated) lesions.

Location	Numbers
Digits (plantar or apical aspect)	16
Plantar Metatarsal Area	20
Plantar Heel	7
Base (Proximal end) of Metatarsal Area	3

Of 45 patients, 69% (21 female and 10 male) demonstrated a complete resolution of verrucae (10 patients with single lesions, 8 with mosaic and 13 with multiple types). There was no significant difference in the cure rates between males and females (*p* = 0.463). Table 2 profiles previous treatments of the resolved and unresolved lesions. Of the 45 patients, 7 (2 single lesions, 2 mosaic lesions and 3 multiple lesions) opted for a second needling treatment as the initial treatment did not fully rid all lesions.

**Table 2:** Previous treatment profile of resolved (R) and unresolved (U) lesions (*n* = 45).

	Salicylic Acid		Cryotherapy		Salicylic Acid & Cryotherapy Combined		Natural and Homeopathic remedies		No previous treatments		Total
	U	R	U	R	U	R	U	R	U	R	
Number	7	12	1	1	3	7	0	2	3	9	45
Percentage %	16	27	2.2	2.2	6.7	15	0	4.4	6.7	20	100

Lesions from 14 patients (31%) failed to resolve. There was no difference in the mean duration of resolved *versus* unresolved lesions (mean duration unresolved 7.67 years ± SD 13.26 *versus* mean resolved 6.68 years ± SD 4.41 (*p* = 0.7571). Three of the patients with unresolved lesions (7%) reported significant reduction of verrucae size and subsequent pain, signifying a clinical improvement. All treated patients reported their pain level after needling as either “none” (*n* = 29 [64%]) or “mild” (*n* = 16 [36%]) describing mild symptoms such as “bruising” or “slight discomfort”. No post-operative infection or scarring was evident on examination or reported by patients post-operatively.

### Discussion

This paper represents the largest case series published to date using Falknor’s method since his original paper was published in 1969. The selection of patients from an adult population with a broad age range (17–66 years) has reduced the possibility of spontaneous regression often observed in child population based studies of this kind. The resolution rate following a maximum of two treatments (69%) indicates a good response when compared to other modalities. Using a similar approach of exposing wart virus to the immune system, Nischal *et al.* [17] auto-implanted debrided wart tissue from sufferers’ feet and implanted into the sub-cutis of the patients forearm or thigh. Analysis of the 27 subjects demonstrated a similar 74% clearance of lesions within three months.

The observed positive clinical outcomes, in this current review, suggest that the hypothesis; provocation of a cell-mediated response as the cause of verrucae regression, is a viable premise. Needling just one lesion often produced a “cascade” effect, whereby the remaining untreated lesions also resolved in a number of patients. Thus, it can be suggested that introducing already HPV infected keratinocytes into the subcutaneous layer appears to facilitate a desired immune response in some patients.

Currently the practice of needling verrucae is not extensively practiced. This may be due to many factors, including lack of published research, preference to utilise traditional and established treatments, (such as salicylic acid and cryotherapy) or simply because many practitioners require update in anaesthesic skills for ankle block infiltration. 

Although the short review period of eight weeks post-operatively, potentially does not rule out completely the possibility of reinfection or recurrence, longer term follow up is required. The authors acknowledge that this is small case series and cannot imply effectiveness and any placebo action inherent cannot be fully determined. However, a larger scale investigation, with objective measures using a control intervention, over a longer period of time would provide a more detailed picture of efficacy and long term immunity of the needling technique.

## 4. Conclusions

Currently, there is no consistently effective treatment for plantar warts. This case series has reviewed the potential for the use of a direct needling technique in the management of plantar warts in an adult population. Following treatment of 45 patients, complete resolution of warts after eight weeks was observed in 31 (69%) cases. This represents a relatively small case series, within a restricted clinical setting. However, a larger scale investigation, with objective measures using a control intervention would provide a more detailed picture of efficacy and long term immunity of this promising technique.

## References

[1] Gibbs S., Harvey I. (2006). Topical treatments for cutaneous warts. Cochrane Database of Systematic Reviews.

[2] Kwok C.S., Holland R., Gibbs S. (2011). Efficacy of topical treatments for cutaneous warts: A meta-analysis and pooled analysis of randomized controlled trials. Br. J. Dermatol..

[3] Lipke M. (2006). An armamentarium of wart treatments. Clin. Med. Res..

[4] Falknor G.W. (1969). Needling—a new technique in verruca therapy. A case report. J. Am. Podiatry Assoc..

[5] Colver G. (2005). Cryosurgery in podiatric practice. Podiatry Now.

[6] Sterling J.C., Handfield-Jones S., Hudson P.M. (2001). Guidelines for the management of cutaneous warts. Br. J. Dermatol..

[7] Moghaddas N. (2004). Periungual verrucae diagnosis and treatment. Clin. Podiatr. Med. Surg..

[8] Frazer I.H. (2009). Interaction of human papillomaviruses with the host immune system: A well evolved relationship. Virology.

[9] Bristow I.R., Stiles C.J. (2012). The treatment of stubborn plantar warts using topical 5% imiquimod cream. Podiatry Rev..

[10] Powell J. (1998). Papillomavirus research and plantar warts. Foot.

[11] Nakayama Y., Asagoe K., Yamauchi A., Yamamoto T., Shirafuji Y., Morizane S., Nakanishi G., Iwatsuki K. (2011). Dendritic cell subsets and immunological milieu in inflammatory human papilloma virus-related skin lesions. J. Dermatol. Sci..

[12] Bruggink S.C., Gussekloo J., Berger M.Y., Zaaijer K., Assendelft W.J., de Waal M.W., Bavinck J.N., Koes B.W., Eekhof J.A. (2010). Cryotherapy with liquid nitrogen *versus* topical salicylic acid application for cutaneous warts in primary care: Randomized controlled trial. CMAJ.

[13] Tyring S. (2000). Immune-response modifiers: A new paradigm in the treatment of human papillomavirus. Curr. Ther. Res..

[14] Parton A.M., Sommerville R.G. (1994). The treatment of plantar verrucae by triggering cell-mediated immunity. J. Br. Pod. Med..

[15] Chapman C., Visaya G. (1998). Treatment of multiple verrucae by triggering cell-mediated immunity—A clinical trial. Br. J. Podiatry.

[16] Skilton B., Mehar Z. (2011). Needling: A treatment option for recalcitrant verrucae pedis. Podiatry Now.

[17] Nischal K.C., Sowmya C.S., Swaroop M.R., Agrawal D.P., Basavaraj H.B., Sathyanarayana B.D. (2012). A novel modification of the autoimplantation therapy for the treatment of multiple, recurrent and palmoplantar warts. J. Cutan. Aesthet. Surg..

